# HNF-4 participates in the hibernation-associated transcriptional regulation of the chipmunk hibernation-related protein gene

**DOI:** 10.1038/srep44279

**Published:** 2017-03-10

**Authors:** Daisuke Tsukamoto, Michihiko Ito, Nobuhiko Takamatsu

**Affiliations:** 1 Kitasato University School of Science, Kanagawa 252-0373, Japan

## Abstract

The chipmunk hibernation-related protein 25 (HP-25) is involved in the circannual control of hibernation in the brain. The liver-specific expression of the *HP-25* gene is repressed in hibernating chipmunks under the control of endogenous circannual rhythms. However, the molecular mechanisms that differentially regulate the *HP-25* gene during the nonhibernation and hibernation seasons are unknown. Here, we show that the hibernation-associated *HP-25* expression is regulated epigenetically. Chromatin immunoprecipitation analyses revealed that significantly less hepatocyte nuclear receptor HNF-4 bound to the *HP-25* gene promoter in the liver of hibernating chipmunks compared to nonhibernating chipmunks. Concurrently in the hibernating chipmunks, coactivators were dissociated from the promoter, and active transcription histone marks on the *HP-25* gene promoter were lost. On the other hand, *small heterodimer partner (SHP)* expression was upregulated in the liver of hibernating chipmunks. Overexpressing SHP in primary hepatocytes prepared from nonhibernating chipmunks caused HNF-4 to dissociate from the *HP-25* gene promoter, and reduced the *HP-25* mRNA level. These results suggest that hibernation-related *HP-25* expression is epigenetically regulated by the binding of HNF-4 to the *HP-25* promoter, and that this binding might be modulated by SHP in hibernating chipmunks.

Of the many physiological seasonal adaptations that occur in animals in response to the physical world, hibernation is one of the most extreme. Mammalian hibernation is a unique circannual physiological adaptation that allows life to be sustained at extremely low body temperatures, which can fall below 10 °C or even 5 °C. During hibernation, heart and breathing rates drop, and the metabolic rate falls to only a few percent of the euthermic level, resulting in a considerable conservation of energy^1^. The mechanistic basis of these dynamic physiological changes in hibernation is not well understood.

The *Tamias asiaticus* (chipmunk) hibernation-related protein 25 is a component in a 140-kDa complex, consisting of HP-20, HP-25, HP-27, and HP-55, that decreases drastically in the blood during hibernation^2^. HP-20, HP-25, and HP-27 contain collagen-like Gly-X-Y repeats near the N terminus and a globular domain in the C terminus, and are highly homologous to each other^2^,^3^. The 140-kDa complex starts decreasing before the onset of hibernation, stays at reduced levels during hibernation, and starts increasing before hibernation ends^2^. Kondo *et al*. showed that the onset of hibernation is marked by an increase in the HP-20c protein complex in the brain; this complex is composed of HP-20, HP-25, and HP-27^4^. Furthermore, hibernation is shortened when an antibody against HP-20c is administered via the lateral ventricles^4^, indicating that HP-20c has a critical role in hibernation.

The *HP-25* gene, which is expressed specifically in the liver, is downregulated in hibernating chipmunks^3^. The *HP-25* mRNA level in the liver fluctuates in parallel with levels of the 140-kDa complex in the blood. Since the levels of the 140-kDa complex fluctuate independently of changes in body temperature^4^, the *HP-25* mRNA expression in the liver is thought to be controlled by endogenous circannual rhythms, rather than by changes in body temperature or by metabolic inhibition at low body temperatures. Thus, elucidating the mechanism of such hibernation-associated endogenous circannual rhythms will shed light on the molecular mechanisms of mammalian hibernation.

Our previous *in vitro* studies revealed that hepatocyte nuclear factor 4 (HNF-4) activates *HP-25* transcription^5^. HNF-4 is a member of the nuclear receptor family^6^. Fatty acids and linoleic acids are assumed to be the endogenous ligands of HNF-4^7^,^8^. HNF-4 is mainly expressed in the liver and binds to direct repeat 1 like motifs as a homodimer^6^,^9^. HNF-4 is a critical transcriptional regulator for many genes specifically expressed in the liver^10^. In addition, HNF-4 interacts with various partner proteins that change its transcriptional activity^11^,^12^. HNF-4 can also recruit several coactivators that modulate chromatin^13^.

In this study, we investigated the mechanism of the hibernation-associated loss of *HP-25* expression in chipmunks. We first analyzed whether the hibernation-associated loss of *HP-25* expression is regulated at the transcriptional level. Subsequently, we focused on the HNF-4-regulated transcriptional activity and on hibernation-associated changes in epigenetic marks on the *HP-25* gene promoter region. Our results suggest that HNF-4 and its coactivators bind the *HP-25* promoter region to epigenetically regulate the *HP-25* transcription changes associated with circannual hibernation.

## Results

### *HP-25* gene transcription is regulated in association with hibernation

**Figure 1:**
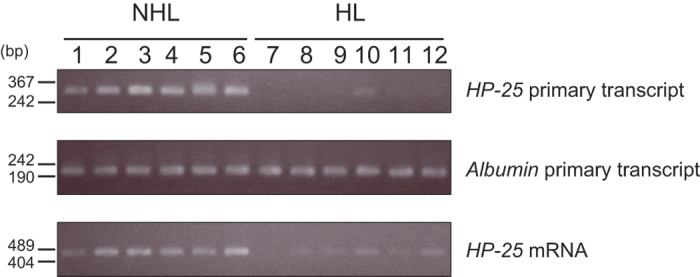
*HP-25* is regulated at the transcriptional level in association with hibernation. *HP-25* primary transcript, *HP-25* mRNA and *albumin* primary transcript were amplified by RT-PCR using total RNA extracted from the liver of nonhibernating (NHL; lanes 1–6) and hibernating chipmunks (HL; lanes 7–12). The PCR products were separated by electrophoresis on a 2% agarose gel.

We previously showed that the chipmunk *HP-25* gene is downregulated in the liver of hibernating chipmunks, based on Northern analysis^3^. However, Northern analysis only indirectly measures gene transcription, since mRNA levels represent the sum of both transcriptional and post-transcriptional events. Here, we verified our initial finding by analyzing the levels of *HP-25* primary transcripts; this approach to determining a gene’s transcriptional state is an alternative to the nuclear run-on assay^14^. Total RNA was isolated from the liver of six nonhibernating chipmunks and six hibernating chipmunks, and the level of *HP-25* primary transcripts was compared by RT-PCR using a pair of primers, one located in exon 1 and the other in intron 1 (Fig. 1 and Supplementary Fig. S1). The PCR amplimers were confirmed to be a single band of the correct size by Southern blot analysis (Supplementary Fig. S1). The *HP-25* mRNA level was also analyzed by RT-PCR using a pair of primers, one located in exon 2 and the other in exon 3. Since the *albumin* mRNA level remains constant regardless of hibernation state^3^, we used the level of *albumin* primary transcripts as a control. The *HP-25* mRNA level was reduced as previously reported^3^, and similarly, the level of *HP-25* primary transcripts was greatly reduced in the hibernating chipmunks compared with nonhibernating chipmunks. Although the *albumin* primary transcript levels differed slightly between individuals, the level was almost constant in nonhibernating and hibernating chipmunks. These results indicate that the *HP-25* gene is in a transcriptionally active state in the liver of nonhibernating chipmunks and shifts to a transcriptionally repressed state in the liver of hibernating chipmunks. This observation was in clear contrast to the constitutively active *albumin* gene.

### Amount of HNF-4 bound to the *HP-25* gene promoter decreased during hibernation

**Figure 2:**
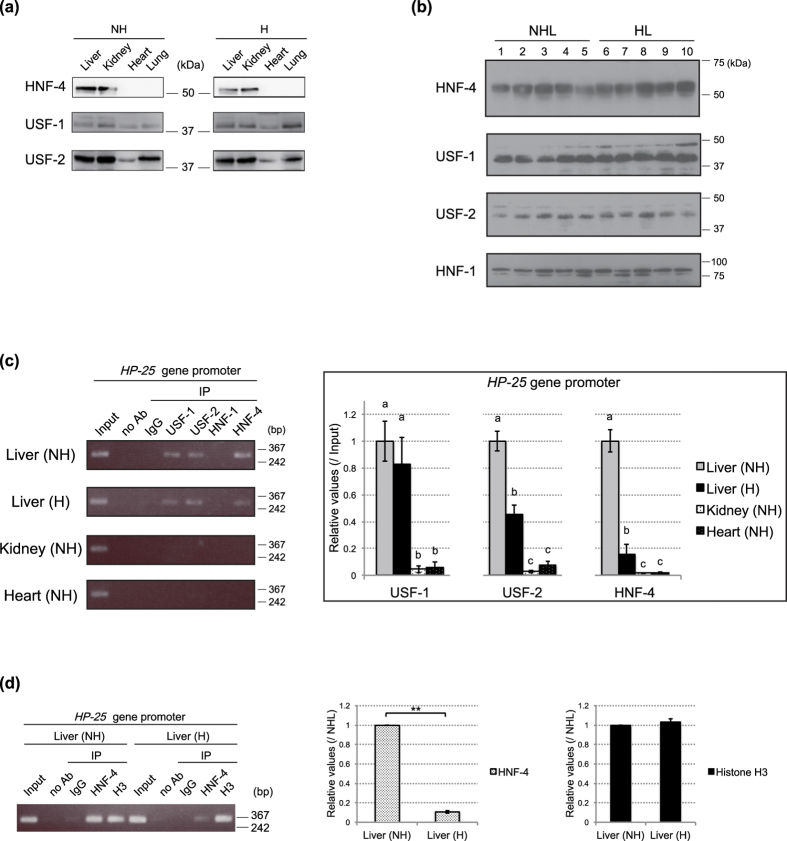
Amount of HNF-4 bound to the *HP-25* promoter increased in the nonhibernation season. (**a**) Immunoblot analyses of HNF-4, USF-1, and USF-2 using nuclear extracts prepared from the liver, kidney, heart, and lung of a nonhibernating (NH) and a hibernating (H) chipmunk. (**b**) Immunoblot analyses of HNF-4, USF-1, USF-2, and HNF-1 were performed as in (**a**) using the liver of five nonhibernating (NHL; lanes 1–5) and five hibernating chipmunks (HL; lanes 6–10). (**c**) Binding of transcription factors to the *HP-25* gene promoter was analyzed by ChIP (left panel) and ChIP-qPCR (right panel). ChIP and ChIP-qPCR were performed with chromatin from the liver, kidney, and heart of a nonhibernating chipmunk (NH) and the liver of a hibernating chipmunk (H) using the indicated antibodies, normal rabbit IgG (IgG), or no antibody (no Ab). Following DNA purification, the samples were analyzed by PCR using primer set specific for the *HP-25* gene promoter region. One hundredth of four percent of the total input sample (Input) was also examined by PCR. Results are representative of more than three each of nonhibernating and hibernating chipmunks. In ChIP-qPCR, the values were normalized to the total input values, and the results are shown as the fold increase over the values for the liver of the nonhibernating chipmunk (NH). Results are means ± SEM for two independent experiments using the samples prepared from different individuals. Different letters (**a–c**) are significantly different at p < 0.05; one-way ANOVA with Tukey-Kramer post test. (**d**) ChIP (left panel) and ChIP-qPCR (middle and right panels) were performed with the indicated antibodies using chromatin from the liver of a nonhibernating chipmunk (NH) and a hibernating chipmunk (H) different from those used in (**c**). In ChIP-qPCR, the values were normalized as in (**c**). Results are means ± SEM for three independent experiments. **p < 0.01; Student’s *t*-test.

Our previous *in vitro* studies revealed that the *HP-25* gene transcription is activated by HNF-4, which is a hepatocyte-enriched transcription factor belonging to the nuclear receptor superfamily, and by upstream stimulatory factor (USF), which is a helix-loop-helix transcription factor^5^,^15^. Here, immunoblotting analyses of nuclear extracts from several tissues of nonhibernating and hibernating chipmunks using antibodies validated for corresponding chipmunk proteins (Supplementary Fig. S2) revealed that HNF-4, USF-1, and USF-2 were present in the kidney, where the *HP-25* gene is not expressed [*HP-25* mRNA was detected only in the liver, but not in the kidney, by northern analysis^3^], as well as in the liver (Fig. 2a). Furthermore, although the amounts of HNF-4 and USF in the liver differed between individuals to some extent, the average amounts were comparable in nonhibernating and hibernating chipmunks (Fig. 2b and Supplementary Fig. S3).

We next used chromatin immunoprecipitation (ChIP) to determine whether HNF-4, USF-1, and USF-2 were involved in the liver-specific transcription of the *HP-25* gene (Fig. 2c). In the kidney, these transcription factors were hardly present on the promoter region of the *HP-25* gene (Fig. 2c). In the liver of nonhibernating chipmunks, USF-1, USF-2, and HNF-4 bound to the *HP-25* promoter region, as expected from our previous results^5^,^15^. To assess whether HNF-4 binds to the *HP-25* gene promoter liver-specifically, we compared HNF-4-binding to the promoter regions of the *HP-25* and *Pklr* genes by ChIP (Supplementary Fig. S4). While the *HP-25* gene is expressed in liver but not in kidney^3^, the *Pklr* gene is expressed in both liver and kidney (Supplementary Fig. S4a). The results showed that HNF-4 bound to the *Pklr* gene promoter in both liver and kidney, but to the *HP-25* gene promoter liver-specifically (Fig. 2c and Supplementary Fig. S4d), consistent with its liver-specific transcription. Of note, USF-1, USF-2, and HNF-4 bound to the *HP-25* gene promoter region even in the liver of hibernating chipmunks (Fig. 2c), where this gene is transcriptionally repressed. However, the amount of HNF-4 bound to the *HP-25* gene promoter region was drastically decreased in hibernating chipmunks (Fig. 2c). In the case of the *albumin* gene, which is transcriptionally upregulated by HNF-1^16^, HNF-1 and Pol II bound the promoter region in the liver of both nonhibernating and hibernating chipmunks (Supplementary Fig. S5), consistent with its constitutively active transcription. ChIP-qPCR showed that about 10 times more HNF-4 was bound to the *HP-25* gene promoter in the liver of nonhibernating compared to hibernating chipmunks (Fig. 2d). On the other hand, the amount of histone H3 on the *HP-25* gene promoter region was almost the same in the liver of both nonhibernating and hibernating chipmunks (Fig. 2d). These findings suggested that the dissociation of HNF-4 from the *HP-25* gene promoter region is one of the major causes of the repression of *HP-25* gene transcription in hibernating chipmunks.

### Histone modifications on the *HP-25* gene promoter region change in association with hibernation

**Figure 3:**
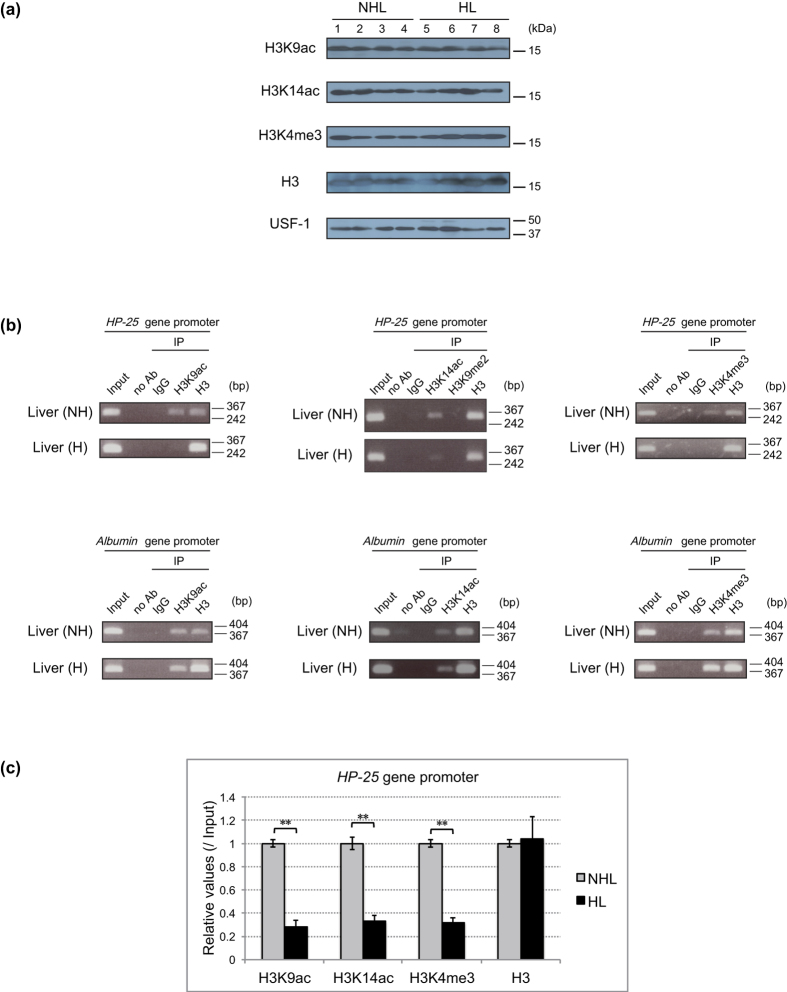
Increased active transcription histone markers on the *HP-25* promoter in the liver of nonhibernating chipmunks. (**a**) Comparison of the amounts of modified histones in the liver of nonhibernating and hibernating chipmunks. Immunoblotting was performed as in Fig. 2b using nuclear extracts from the liver of four nonhibernating chipmunks (NHL; lanes 1–4) and four hibernating chipmunks (HL; lanes 5–8). USF-1 was used as a loading control. (**b**) ChIP was performed as in Fig. 2c with chromatin from the liver of a nonhibernating chipmunk (NH) and a hibernating chipmunk (H) using the indicated antibodies and primer sets specific for the indicated gene promoter regions. (**c**) ChIP-qPCR was performed as in Fig. 2d with chromatin from the liver of a nonhibernating (NHL) and a hibernating chipmunk (HL) using the indicated antibodies. Results are means ± SEM for three independent experiments performed in technical triplicate. **p < 0.01. The chipmunks used in (**b**) and (**c**) are different individuals.

The acetylation and methylation of histone at certain residues, such as the trimethylation of histone H3 at lysine (K) 4 and the acetylation at K9 and K14, are generally involved in gene activation, whereas the lack of histone acetylation is a hallmark of gene repression^17^,^18^,^19^,^20^. Histone modifications for active transcription destabilize higher-order chromatin structures and render nucleosomal DNA more accessible to transcription factors^21^. To examine the involvement of epigenetic regulation in *HP-25* gene transcription, we first compared the total amount of histone H3 acetylated at K9 and K14 and trimethylated at K4 in the liver between nonhibernating and hibernating chipmunks by immunoblotting (Fig. 3a). The total amounts of the modified histones in the liver were almost equal between nonhibernating and hibernating chipmunks. We then analyzed the modification states of the histone H3 specifically bound to the *HP-25* gene promoter region by ChIP (Fig. 3b,c). Histone H3 bound to the *HP-25* promoter region was significantly more acetylated at K9 and K14 and more trimethylated at K4 in the liver of nonhibernating chipmunks than in hibernating chipmunks (Fig. 3b upper part and 3c), but H3K9 dimethylation, one of the repressive histone modifications, was not detected even in hibernating chipmunks (Fig. 3b middle upper part). On the other hand, the histone H3 bound to the *albumin* promoter region was almost equally acetylated at K9 and K14 and trimethylated at K4 in the liver of nonhibernating and hibernating chipmunks (Fig. 3b lower part). These results were consistent with our findings on the transcriptional states of the *HP-25* and *albumin* genes (Fig. 1). Considering that the total amounts of the modified histones were similar between nonhibernating and hibernating chipmunks (Fig. 3a), these histone modifications are probably regulated at the promoter region of specific genes involved in hibernation, including at least the *HP-25* gene.

### HNF-4 and coactivators activate *HP-25* transcription in the liver of nonhibernating chipmunks

**Figure 4:**
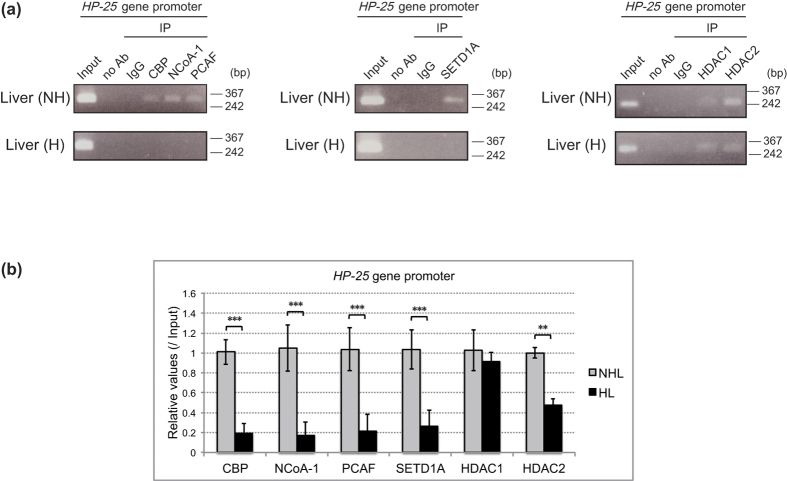
Coactivators enhance the *HP-25* transcriptional activation in nonhibernating chipmunks. (**a**) ChIP was performed as in Fig. 2c using the indicated antibodies. (**b**) ChIP-qPCR was performed as in Fig. 2d with chromatin from the liver of two nonhibernating (NHL) and two hibernating chipmunks (HL) using the indicated antibodies. Results are means ± SEM for two independent experiments performed in technical triplicate. **p < 0.01, ***p < 0.001. The chipmunks used in (**a**) and (**b**) are different individuals.

To elucidate the mechanism of the hibernation-associated changes in histone modifications on the *HP-25* gene promoter, we used ChIP to analyze the histone acetyltransferases (HATs), histone methyltransferases (HMTs), and histone deacetylases (HDACs) bound to the *HP-25* promoter region (Fig. 4a,b). Histone H3 can be acetylated at K9 and K14 by HATs such as CBP, NCoA-1, PCAF, p300, GCN5, MOZ, MORF, and TFIIIC, and can be methylated at K4 by HMTs such as SETD1A, SETD1B, MLL1, MLL2, and ASH1L^17^,^19^,^22^,^23^. HDACs are classified into groups I–IV, and the class I HDACs HDAC1, HDAC2, HDAC3, and HDAC8 generally function as subunits in multiprotein complexes that are crucial for repressing transcription^24^. ChIP analyses revealed that HDAC1 and HDAC2 bound the *HP-25* promoter region in the liver of both nonhibernating and hibernating chipmunks (Fig. 4a right panel and 4b). In contrast, CBP, NCoA-1, PCAF, and SETD1A were found on the *HP-25* gene promoter mainly in the liver of nonhibernating chipmunks (Fig. 4a left and middle panels and 4b). Thus, hibernation-associated histone modifications are likely to be modulated by HAT and HMT, which are associated with the *HP-25* gene promoter in nonhibernating chipmunks and dissociated from the promoter region in hibernating chipmunks.

To investigate the involvement of HATs and HMT(s) in activating the *HP-25* gene transcription, we transiently transfected HeLa cells with an *HP-25* gene promoter-reporter plasmid and expression plasmids for CBP, SETD1A, and HNF-4 singly (Supplementary Fig. S6a) or in combination (Supplementary Fig. S6b). Although neither CBP nor SETD1A alone could activate the *HP-25* transcription, both could facilitate its transcriptional activation by HNF-4, and the combination of CBP and SETD1A further enhanced the *HP-25* transcription (Supplementary Fig. S6a,b). CBP’s enhancement of the *HP-25* transcriptional activation was repressed by curcumin, a specific inhibitor of CBP HAT activity^25^, in a dose-dependent manner (Supplementary Fig. S6c). These results indicate that the *HP-25* gene transcription is activated by HNF-4 in combination with HATs and HMT(s) in the liver of nonhibernating chipmunks.

### SHP upregulation represses *HP-25* transcription by suppressing HNF-4 activity during hibernation

**Figure 5:**
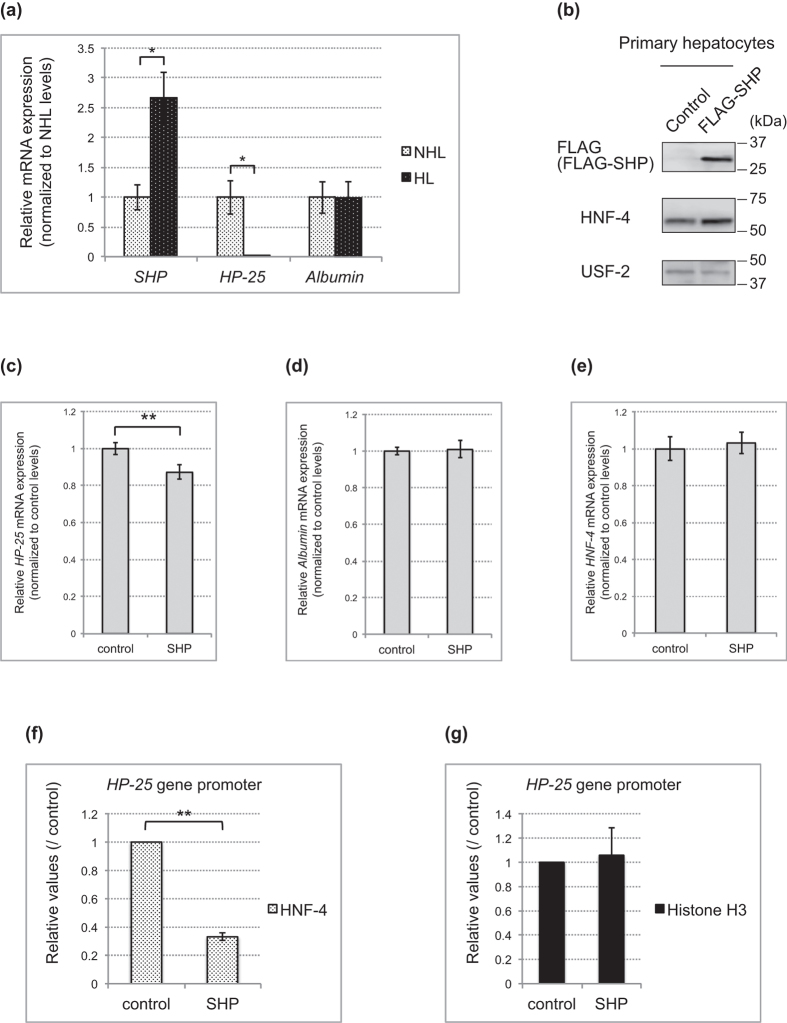
SHP represses *HP-25* gene transcription by inhibiting HNF-4 binding to the promoter. (**a**) *SHP, HP-25*, and *albumin* mRNA levels in the liver of four nonhibernating chipmunks (NHL) and four hibernating chipmunks (HL), measured by RT-qPCR. The results were normalized to the amount of mRNA of the respective genes in the NHL. The results are shown as means ± SEM. *p < 0.05. (**b**–**g**) Primary hepatocytes prepared from a nonhibernating chipmunk were transfected with a FLAG-SHP expression plasmid or an empty vector plasmid (control). In (**b**), whole-cell extracts from the primary hepatocytes were immunoblotted with the indicated antibodies. USF-2 was used as a loading control. In (**c**–**e**), total RNA was prepared from the primary hepatocytes (SHP or control), and the endogenous *HP-25* (**c**), *albumin* (**d**), and *HNF-4* (**e**) expression was assessed by RT-qPCR. The results were normalized to the amount of mRNA for each gene in control cells. Results show means ± SEM for three independent experiments. **p < 0.01. In (f and g), ChIP-qPCR was performed with chromatin from the primary hepatocytes using anti-HNF-4 (**f**) or anti-histone H3 (**g**) antibodies, and the values were normalized to the total input value. Results are shown as the fold increase over the control value. Results show means ± SEM for three independent experiments. **p < 0.01.

Our results thus far indicated that the *HP-25* transcription was probably repressed in hibernating chipmunks due to reduced levels of HNF-4 bound to the promoter (Fig. 2d) and the dissociation of coactivators from the promoter (Fig. 4a,b). Although several microRNAs have been implicated in the translational repression of HNF-4 expression^26^,^27^, the observation that the HNF-4 levels were unchanged in nonhibernating and hibernating chipmunks would exclude microRNAs from the cause of this repression. Small heterodimer partner (SHP), which belongs to a group of atypical nuclear receptors that lack a classical DNA-binding domain and are corepressors of a number of nuclear receptors^28^, represses HNF-4 activity by competing with the coactivator SRC-3^29^. We could not detect endogenous SHP protein expression in the liver with any of several commercial anti-SHP antibodies, so we used RT-qPCR to compare *SHP* expression in the liver of nonhibernating and hibernating chipmunks (Fig. 5a). Notably, unlike the *HP-25* mRNA levels, the amount of *SHP* mRNA was about 2.5-fold higher in hibernating than in nonhibernating chipmunks. To examine whether chipmunk SHP affects the transcriptional activity of HNF-4, we conducted a transient transfection assay using an *HP-25* gene promoter-reporter plasmid and expression plasmids for HNF-4, CBP, and SHP singly (Supplementary Fig. S6d) or in combination (Supplementary Fig. S6e), in HeLa cells. We found that SHP repressed *HP-25* gene transcription activated by HNF-4 alone or in combination with CBP (Supplementary Fig. S6d,e). We next examined interactions between chipmunk HNF-4 and chipmunk SHP in cultured cells. In HeLa cells transfected with chipmunk HNF-4 and FLAG-tagged chipmunk SHP, immunocytochemistry using fluorescent and confocal microscopies showed that significant portions of HNF-4 and SHP were colocalized in nucleus (Supplementary Fig. S6f), and HNF-4 and SHP were co-precipitated in a co-immunoprecipitation assay (Supplementary Fig. S6g). Taken together, these results indicate that chipmunk SHP interacts with chipmunk HNF-4 in the nucleus and represses the *HP-25* gene transcription activated by HNF-4.

Finally, to examine the effect of ectopic SHP expression on endogenous *HP-25* gene expression, we transfected a FLAG-tagged SHP-expression construct into primary hepatocytes prepared from a nonhibernating chipmunk. The expression of FLAG-tagged SHP was verified by immunoblotting (Fig. 5b). RT-qPCR analysis revealed that although SHP overexpression did not affect the *albumin* mRNA levels, the *HP-25* mRNA was reduced by 15% (Fig. 5c,d). Ectopic SHP expression did not affect the amount of HNF-4 protein or *HNF-4* mRNA (Fig. 5b,e). ChIP-qPCR revealed that SHP overexpression decreased the amount of HNF-4 bound to the *HP-25* gene promoter by about 70% (Fig. 5f), although the amount of histone H3 on the *HP-25* gene promoter region was almost the same with or without SHP transfection (Fig. 5g). Taken together, these results indicate that SHP expression is upregulated in hibernating chipmunks and that SHP represses *HP-25* expression by removing HNF-4 from the promoter.

## Discussion

Mammalian hibernation is accompanied by various physiological changes^1^ that are assumed to be under genetic control. Although there is growing evidence for differential gene expression during hibernation^30^,^31^,^32^,^33^,^34^,^35^,^36^,^37^,^38^, the molecular mechanisms underlying hibernation-associated gene regulation are unknown. In this study, we found that differential *HP-25* transcription in the liver between nonhibernating and hibernating chipmunks is epigenetically regulated: H3K9 and K14 were highly acetylated and H3K4 was highly trimethylated in the *HP-25* promoter region in nonhibernating chipmunks, but not in hibernating chipmunks. There was a positive correlation among HNF-4 binding, HAT/HMT binding, and histone acetylation/trimethylation levels. Notably, SHP, which negatively regulates HNF-4, was upregulated in the liver of hibernating chipmunks, and ectopically expressing SHP in primary hepatocytes prepared from nonhibernating chipmunks resulted in the dissociation of HNF-4 from the *HP-25* promoter and the repression of *HP-25* gene transcription.

Our ChIP analyses revealed that in tissues like the kidney, where *HP-25* is not expressed, the transcription factors HNF-4, USF-1, and USF-2 were present but hardly bound to the *HP-25* promoter region (Fig. 2). These results indicate that the liver-specific transcription of the *HP-25* gene is established by assembling a higher order chromatin structure around the promoter region, which is inaccessible by transcription-factors, in nonexpressing tissues. Our previous finding that USF binding to the CACGTG sequence in the *HP-27* promoter region is inhibited by CpG methylation in nonexpressing tissues supports this idea^39^. On the other hand, in the liver of hibernating chipmunks, where *HP-25* is transcriptionally repressed, the transcription factors HNF-4, USF-1, and USF-2 still bound the *HP-25* gene promoter (Fig. 2), but a repressive histone mark was not observed (Fig. 3b), indicating that the chromatin structures surrounding the *HP-25* gene do not change so drastically that they prevent transcription factors from binding, unlike in nonexpressing tissues like the kidney.

HDACs are traditionally considered to be transcriptional corepressors^24^. However, HDAC1 and HDAC2 were found on the *HP-25* promoter region in the liver of nonhibernating chipmunks (Fig. 4a,b). Recent genome-wide mapping revealed that HDACs localize to many active genes^40^,^41^. These observations support the idea that HDACs are recruited to active genes to reset the chromatin modification states and maintain an adequate level of histone acetylation^40^,^42^, and our result is consistent with this idea. On the other hand, HDACs are also thought to enforce the hypo-acetylated chromatin state of the *HP-25* gene in hibernating chipmunks (Fig. 4a,b).

In this study, we showed that the transcriptional repression of the *HP-25* gene in hibernating chipmunks results mainly from the dissociation of HNF-4 from the promoter, possibly due to SHP, which is upregulated during hibernation. SHP is proposed to repress gene transcription by interacting with other nuclear receptors via two dimerization LXXLL-related motifs in a ligand-binding domain^43^, or by binding to the C-terminal activation function-2 helix of other nuclear receptors through these motifs^28^. Since chipmunk SHP also possesses both LXXLL-related motifs in a ligand-binding domain, chipmunk SHP probably interacts with chipmunk HNF-4 through these two functional motifs. SHP is also reported to interact with lysine-specific histone demethylase 1 (LSD1)^44^,^45^, which is associated with a CoREST–SIRT1 repressor complex that removes H3K4 mono-, di- and trimethylation marks^45^. These reports imply that SHP might also directly repress *HP-25* transcription by recruiting histone modification enzymes to the *HP-25* gene promoter via an interaction with HNF-4 bound to the promoter.

**Figure 6:**
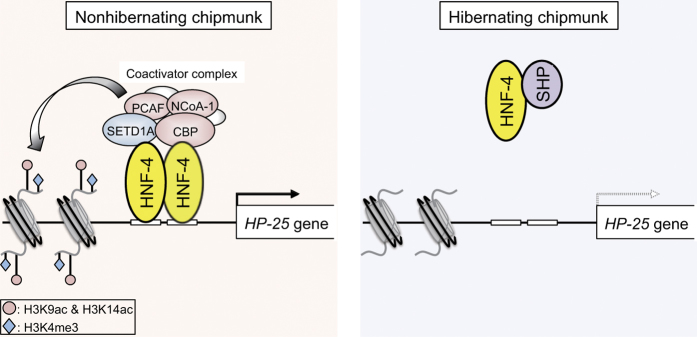
A schematic model of the hibernation-associated transcriptional regulation of the *HP-25* gene based on this study. In nonhibernating chipmunks, HNF-4 binds the *HP-25* gene promoter region to activate *HP-25* transcription in combination with coactivators and active histone modification marks (left side part). On the other hand, in hibernating chipmunks, HNF-4 and its coactivators dissociate from the promoter, possibly by an interaction of HNF-4 with SHP whose expression is upregulated during hibernation, and active transcription histone marks are removed, resulting in transcriptional repression of the *HP-25* gene (right side part).

The *HP-25* gene expression in the liver is thought to be regulated by the endogenous circannual rhythm responsible for hibernation. Our results suggest that this hibernation-associated regulation of *HP-25* transcription occurs as follows: in the liver of nonhibernating chipmunks, HNF-4 binds the *HP-25* promoter region to activate *HP-25* transcription in combination with coactivators and active histone modification marks; in hibernating chipmunks, HNF-4 and its coactivators dissociate from the *HP-25* gene promoter, possibly by an interaction with SHP (which is now upregulated), and active transcription histone marks are removed from the promoter region (Fig. 6). Since transcription and translation are drastically decreased at low body temperatures^46^,^47^,^48^, these repression processes are likely to occur before or during the entry into hibernation. On the other hand, a reduction in SHP during the nonhibernation season would allow HNF-4 to bind to the *HP-25* promoter, which could facilitate H3K4 trimethylation and histone acetylation on the promoter region by recruiting HMTs and HATs, leading to *HP-25* transcriptional activation. Together, controlled HNF-4 binding to the *HP-25* gene promoter is likely to play a key role in the differential regulation of *HP-25* transcription between the nonhibernating and hibernating chipmunks. Recently, it has been reported that not only hibernating mammals but also non-hibernating mammals contain *HP-20, HP-25* and *HP-27* genes, and in cow, whose *hibernation-related protein (HP*) genes are expressed specifically in the liver, circulating levels of HP-25 and HP-27 in the blood and cerebrospinal fluid change seasonally as is the case with the chipmunk HPs^49^,^50^. Even when chipmunks were kept under conditions of constant warmth (23 °C) with a 12 h:12 h light:dark period cycle, the HP level in the blood and their gene expression in the liver still showed seasonal oscillations similar to those in animals with a low body temperature (Tb) during hibernation season, indicating that the gene expression of HPs is regulated by endogenous circannual rhythms, and independent of Tb changes^4^. Since there is a HNF-4 binding site in the cow *HP-25* gene promoter (data not shown), transcription of the cow *HP-25* gene may be regulated by a general nuclear receptor HNF-4 as the chipmunk *HP-25* gene. Although it has not been established that cow HPs oscillate in an endogenously generated circannual manner^49^, the gene regulation of HPs is likely to be under the control of a circannual clock common to chipmunk and cow. In recent years, the presence of a circannual pacemaker in the pars tuberalis of the pituitary gland has been suggested^51^,^52^. Identifying the repressors that regulate *HP-25* transcription and HNF-4’s binding to the *HP-25* promoter poses an interesting challenge. Based on our results, elucidating the upstream factors that control the epigenetic regulation of *HP-25* transcription will help to identify the circannual signals from the circannual pacemaker common to hibernators and nonhibernators. Also, comparative analyses of the pathways affecting the *HP-25* gene transcriptional regulation between hibernators and nonhibernators, together with functional analysis of the HP-25 protein, may lead to identification of hibernation signals.

## Material and Methods

### Ethics statement

All recombinant DNA experiments in this study strictly followed a protocol and guidelines approved by the Institutional Recombinant DNA Experiment Safety Committee of Kitasato University.

### Animals

Male chipmunks (*Tamias asiaticus*) purchased from Pet Easy Space (Osaka) were individually housed and offered standard rodent chow and water *ad libitum*. The chipmunks were kept at 23 °C with a 12 h:12 h light:dark period during the nonhibernation season (April–September) and at 5 °C in darkness during the hibernation season (October–March). Nonhibernating chipmunks were summer-active animals sampled in June or July. Hibernating chipmunks were sampled approximately three months after first immergence into torpor. The topor bouts usually last 5–6 days. At the sampling time, these hibernating chipmunks had been hibernating for 3–4 days: they were torpid, and their body surface temperatures were under 10 °C measured by an infrared thermometer. All animals were deeply anesthetized with isoflurane prior to sacrifice. Tissues were immediately excised, frozen in liquid nitrogen, and stored at −80 °C until use. All of the protocols were in accordance with the guidelines of the Institutional Animal Care and Use Committee of Kitasato University, and all experimental procedures were approved by the same committee.

### RT-PCR of primary transcripts

Total RNA prepared from the liver using the RNeasy Mini Kit (Qiagen) was treated with DNase I using the TURBO DNA-free kit (Ambion). The reverse transcription of total RNA and subsequent PCR were performed using the mRNA Selective PCR Kit (Takara) to amplify the *HP-25* primary transcript, *HP-25* mRNA, and *albumin* primary transcript. The PCR products were fractionated using a 2% agarose gel. The following primer sets were used for PCR: CM25 + 114F_exon1, 5′-ATGCCTGCACAAAGAGG-3′, and CM25 + 409R_intron1, 5′-GGCCTGATATCATTCCTCC-3′; cmHP25 + 216F_exon2, 5′-AATTCTGAACCCTGTGGACCT-3′, and cmHP25 + 683R_exon3, 5′-TTCCAGCCAGACTTTGTCCC-3′; CMALB + 868F_exon2, 5′-GGGAGAGCAAAATTTTAAAGG-3′, and CMALB + 1066R_intron2, 5′-ACCAGTGGCTACCATATTAGA-3′.

### Immunoblot analysis

Nuclear extracts were prepared from the liver, kidney, heart, and lung of nonhibernating and hibernating chipmunks^39^. Immunoblotting was performed as described previously^39^. The anti-USF-1 (sc-229) and anti-USF-2 (sc-862) antibodies were purchased from Santa Cruz Biotechnology. The anti-HNF-4 antibodies (sc-8987 and H1415) were purchased from Santa Cruz Biotechnology and Perseus Proteomics, respectively. The anti-HNF-1 antibody (H69220) was purchased from Transduction Laboratories. The anti-acetyl-histone H3 (Lys9) (07-352) and anti-acetyl-histone H3 (Lys14) (07-353) antibodies were purchased from Millipore. The anti-histone H3 (tri methyl K4) (ab8580) and anti-histone H3 (ab1791) antibodies were purchased from Abcam. The anti-DYKDDDDK antibody (KO602) was purchased from TransGenic Inc.

### ChIP analysis

Chromatin immunoprecipitation (ChIP) was performed as described previously^15^. All ChIP experiments were performed three or more times using independent chromatin preparations. The normal rabbit IgG (sc-2027), and antibodies against HNF-4 (sc-8987), HNF-1 (sc-8986), Pol II (sc-9001), NCoA-1 (sc-8995), and PCAF (sc-8999) were purchased from Santa Cruz Biotechnology. The anti-dimethyl-histone H3 (Lys9) antibody (07-441) was purchased from Millipore. The anti-HDAC1 (ab7028), anti-HDAC2 (ab7029), and anti-CBP (ab2832) antibodies were purchased from Abcam. The anti-SETD1A antibody (NB100–559A) was purchased from Novus Biologicals. PCR was carried out with the following primer sets: CM25-230F, 5′-TCTTGCTCTGATGGTTTGTTGACAC-3′, and CM25 + 79 R, 5′-GCCTCTCTCCTCTTTAGTTTTCTTC-3′, which amplicon size is 310 bp; CMALB-380F, 5′-ATTTCCAAGCAGAATCATTGG-3′, and CMALB-11R, 5′-ATAGAAAAGGTAGGATGAATG-3′, which amplicon size is 392 bp. ChIP quantitative PCR (ChIP-qPCR) was performed with the indicated number of samples in technical triplicate using SsoFast EvaGreen Supermix (Bio-Rad) with the following primer set: CM25-166F, 5′-GTGACCACGTAAGGGGCAGG-3′, and CM25-24R, 5′-CTGATCTTTCCTGAGGTCAGTGTC-3′, which primer set was more efficient and selective in SsoFast PCR amplification. Both *HP-25* gene primer sets, CM25-230F and CM25 + 79 R, and CM25-166F and CM25-24R, can amplify the regions encompassing the USF-1, USF-2, and HNF-4 binding sites. The PCR amplimers were confirmed to be a single band of the correct size by agarose gel electrophoresis. ChIP-qPCR data were normalized by the % input method and were shown as the fold increase over the value in the nonhibernating chipmunk liver. Results are expressed as means ± SEM of the indicated number of independent determinations.

### Transfection and luciferase assays

The promoter-reporter plasmid construction of pCM25G-260/luc was described previously^5^. HeLa cells were cultured as described previously^5^. Cells were plated at 4 × 10^4^ cells in a 24-well plate. After 24 h, the cells were transfected with 400 ng of a firefly luciferase promoter-reporter plasmid, and with 10 ng of the *Renilla* luciferase internal control plasmid pRL-SV40 (Promega), using TransIT-LT1 reagent (Mirus). Where denoted, the cells were cotransfected with 100 ng of the mouse CBP expression construct pcDNA3/mCBP, 100 ng of the mouse SETD1A expression construct pcDNA3/mSETD1A, 5 ng of the chipmunk HNF-4 expression construct pcDNA3/cmHNF-4, and/or 100 ng of the chipmunk SHP expression construct pcDNA3/FLAG-cmSHP. After 24 h, the luciferase activity was measured using the Dual-Luciferase Reporter Assay System (Promega). Where denoted, curcumin (Sigma) was added at the indicated concentrations 12 h after transfection, and the luciferase activity was measured after another 12 h.

### Immunocytochemistry

HeLa cells were grown on uncoated glass coverslips and transfected with pcDNA3/cmHNF-4 and pcDNA3/FLAG-cmSHP using Polyethylenimine Max (Polysciences, Inc.). At 24 h after transfection, the cells were fixed with 10% Formaldehyde Neutral Buffer Solution (Nacalai Tesque) for 10 min, mounted on glass slides, and observed with a BZ-8100 fluorescence microscope (Keyence) or an LSM510 confocal laser-scanning microscope (Zeiss). To detect HNF-4 and FLAG-SHP, the cells were incubated with an anti-HNF-4 antibody and anti-DYKDDDDK antibody overnight at 4 °C, washed three times with 0.5% BSA in PBS, and incubated with Alexa Fluor 488 goat anti-rabbit IgG and Alexa Fluor 594 goat anti-mouse IgG (Thermo Scientific) for 1 h at room temperature. To detect nuclei using a fluorescence microscope, the cells were washed twice with PBS and stained with 1 μg/ml Hoechst 33258 (Thermo Scientific) solution for 10 min at room temperature. The cells were then washed with H_2_O, mounted on a slide, and observed under the microscope.

### Co-immunoprecipitation Assays

HeLa cells were plated on a 10-cm dish, and transfected with pcDNA3/cmHNF-4 and pcDNA3/FLAG-cmSHP using Polyethylenimine Max (Polysciences, Inc.). At 48 h after transfection, the cells were lysed in RIPA buffer (50 mM Tris-HCl pH 8.0, 150 mM NaCl, 1% NP-40, 0.5% sodium deoxycholate, 0.1% SDS, and protease inhibitor cocktails) for 30 min at 4 °C. The lysates were centrifuged at 12,000 x *g* for 30 min at 4 °C. The supernatants were collected and incubated with Ab-Capcher (ProteNova) and IgG. The pre-cleared samples were centrifuged, and the supernatants were collected. The pre-cleared supernatants were incubated with an anti-HNF-4 antibody, anti-FLAG M5 antibody (Sigma Aldrich), or pre-immune IgG (used as a negative control) at 4 °C overnight. Immunoprecipitated samples were then captured by Ab-Capcher. The Ab-Capcher beads were washed three times with wash buffer (10 mM Tris-HCl pH 7.5, 1 mM EDTA pH 8.0, 150 mM NaCl, 1% Triton X-100, and protease inhibitor cocktail), and the immunoprecipitated proteins were eluted with SDS-PAGE loading buffer. The eluates were analyzed by immunoblotting using an anti-HNF-4 or anti-DYKDDDDK antibody.

### Reverse transcription qPCR (RT-qPCR)

Total RNA was obtained from chipmunk livers and primary hepatocytes using ISOGEN II (Nippon Gene). First-strand cDNA was synthesized using the PrimeScript 1st Strand cDNA Synthesis Kit (Takara) with random primers following the manufacturer’s instructions; qPCR was performed with four samples in technical triplicate using the SYBR Green Realtime PCR Master Mix (Toyobo) and a Rotor-Gene Q apparatus (Qiagen). The following PCR primers were used: cmSHP + 490 F, 5′-CTGGGCCCCAAAGAGTATGC-3′, and cmSHP + 572 R, 5′-ATGTGGGAGGAGGCATGAAG-3′; cmHP25 + 607 F, 5′-AAGCCAATGACAGCTACAAACAT-3′, and cmHP25 + 799 R, 5′-TTCAGAATTACAAGAGGGATGGA-3′; cmALB + 502 F, 5′-AGAAAATAACCTGAGGCTTTTGG-3′, and cmALB + 675 R, 5′-TTCATAGCCTCAATCTTTGGTGT-3′; cmACTB + 226 F, 5′-GTCACCAACTGGGACGACAT-3′, and cmACTB + 401 R, 5′-ACATACATGGCTGGGGTGT-3′. Beta-actin (ACTB) was used as an endogenous control to normalize gene expression. Relative mRNA expression levels were presented as means ± SEM. Statistical differences were analyzed by Student’s *t*-test.

### Primary hepatocyte cultures and transfection

Primary hepatocytes of nonhibernating chipmunks were obtained by the conventional collagenase method with minor modifications. Following liver perfusion with collagenase type IV (Worthington), the dispersed cells were filtered successively through 500-μm and 125-μm stainless steel sieves, and washed with DMEM four times. In all experiments, the hepatocytes were more than 80% viable as judged by trypan blue exclusion. After centrifugation, the cell pellet was resuspended in Williams’ medium E supplemented with 10% FCS, 1 mM insulin, 1 mM dexamethasone, 100 U/ml penicillin, 0.1 mg/ml streptomycin, 0.5 mg/ml fungizone, and 0.1 mg/ml kanamycin, and the hepatocytes were plated on collagen-coated BioCoat dishes (Corning). On the next day, pcDNA3/FLAG-cmSHP or pcDNA3/FLAG were transfected into the hepatocytes using Lipofectamine 2000 (Invitrogen) following the manufacturer’s instructions. The hepatocytes were cultured for 2 additional days, and then whole-cell extracts and total RNA were prepared.

### Statistical analysis

Student’s *t*-test (2-tailed 2, type 2), Tukey-Kramer Multiple Comparison test, one-way ANOVA, or two-way ANOVA were used to compare the difference from the control group. When the interaction was significant at p < 0.05 by one-way ANOVA or two-way ANOVA, post hoc multiple comparison was made using Tukey-Kramer test. Two-way ANOVA was also used to analyze the interaction between treatments.

## Additional Information

**Accession codes**: The accession number for the chipmunk SHP sequence reported in this paper is Genbank: LC144633.

**How to cite this article:** Tsukamoto, D. *et al*. HNF-4 participates in the hibernation-associated transcriptional regulation of the chipmunk hibernation-related protein gene. *Sci. Rep.* **7**, 44279; doi: 10.1038/srep44279 (2017).

**Publisher's note:** Springer Nature remains neutral with regard to jurisdictional claims in published maps and institutional affiliations.

## Supplementary Material

Supplementary Information

## References

[b1] Wang L. C. H. & Lee T. F. Topor and Hibernation in Mammals: Metabolic, Physiological, and Biochemical Adaptations. In Comprehensive Physiology 507–532 (American Physiological Society, 2011).

[b2] Kondo N. & Kondo J. Identification of novel blood proteins specific for mammalian hibernation. J. Biol. Chem. 267, 473–478 (1992). 1730610

[b3] Takamatsu N., Ohba K., Kondo J., Kondo N. & Shiba T. Hibernation-associated gene-regulation of plasma-proteins with a collagen-like domain in mammalian hibernators. Mol. Cell. Biol. 13, 1516–1521 (1993). 8441393 10.1128/mcb.13.3.1516-1521.1993 PMC359463

[b4] Kondo N. . Circannual control of hibernation by HP complex in the brain. Cell 125, 161–172, doi: 10.1016/j.cell.2006.03.017 (2006). 16615897

[b5] Kojima M., Takamatsu N., Ishii T., Kondo N. & Shiba T. HNF-4 plays a pivotal role in the liver-specific transcription of thechipmunk HP-25 gene. Eur. J. Biochem. 267, 4635–4641, doi: 10.1046/j.1432-1327.2000.01499.x (2000). 10903495

[b6] Sladek F. M., Zhong W. M., Lai E. & Darnell J. E. Liver-enriched transcription factor HNF-4 is a novel member of the steroid-hormone receptor superfamily. Genes. Dev. 4, 2353–2365, doi: 10.1101/gad.4.12b.2353 (1990). 2279702

[b7] Wisely G. B. . Hepatocyte nuclear factor 4 is a transcription factor that constitutively binds fatty acids. Structure 10, 1225–1234 (2002). 12220494 10.1016/s0969-2126(02)00829-8

[b8] Yuan X. H. . Identification of an Endogenous Ligand Bound to a Native Orphan Nuclear Receptor. Plos One 4, e5609, doi: 10.1371/journal.pone.0005609 (2009). 19440305 PMC2680617

[b9] Jiang G. Q., Nepomuceno L., Hopkins K. & Sladek F. M. Exclusive homodimerization of the orphan receptor hepatocyte nuclear factor-4 defines a new subclass of nuclear receptors. Mol. Cell. Biol. 15, 5131–5143 (1995). 7651430 10.1128/mcb.15.9.5131 PMC230760

[b10] Watt A. J., Garrison W. D. & Duncan S. A. HNF4: A central regulator of hepatocyte differentiation and function. Hepatology 37, 1249–1253, doi: 10.1053/jhep.2003.50273 (2003). 12774000

[b11] Peng H., Zhu Q. S., Zhong S. P. & Levy D. Transcription of the human microsomal epoxide hydrolase gene (EPHX1) is regulated by an HNF-4 alpha/CAR/RXR/PSF complex. Biochim. Biophys. Acta 1829, 1000–1009, doi: 10.1016/j.bbagrm.2013.05.003 (2013). 23714182

[b12] Zhang Y. X., Bonzo J. A., Gonzalez F. J. & Wang L. Diurnal Regulation of the Early Growth Response 1 (Egr-1) Protein Expression by Hepatocyte Nuclear Factor 4 alpha (HNF4 alpha) and Small Heterodimer Partner (SHP) Cross-talk in Liver Fibrosis. J. Biol. Chem. 286, 29635–29643, doi: 10.1074/jbc.M111.253039 (2011). 21725089 PMC3191004

[b13] Yoshida E. . Functional association between CBP and HNF4 in trans-activation. Biochem. Biophys. Res. Commun. 241, 664–669, doi: 10.1006/bbrc.1997.7871 (1997). 9434765

[b14] Elferink C. J. & Reiners J. J. Quantitative RT-PCR on CYP1A1 heterogeneous nuclear RNA: A surrogate for the *in vitro* transcription run-on assay. Biotechniques 20, 470–477 (1996). 8679208 10.2144/19962003470

[b15] Tsukamoto D. . USF is involved in the transcriptional regulation of the chipmunk HP-25 gene. Gene 396, 268–272, doi: 10.1016/j.gene.2007.03.014 (2007). 17467197

[b16] Maire P., Wuarin J. & Schibler U. The role of cis-acting promoter elements in tissue-specific albumin gene expression. Science 244, 343–346 (1989). 2711183 10.1126/science.2711183

[b17] Kouzarides T. Chromatin modifications and their function. Cell 128, 693–705, doi: 10.1016/j.cell.2007.02.005 (2007). 17320507

[b18] Cheung P. & Lau P. Epigenetic regulation by histone methylation and histone variants. Mol. Endocrinol. 19, 563–573, doi: 10.1210/me.2004-0496 (2005). 15677708

[b19] Sterner D. E. & Berger S. L. Acetylation of histones and transcription-related factors. Microbiol. Mol. Biol. Rev. 64, 435–459, doi: 10.1128/mmbr.64.2.435-459.2000 (2000). 10839822 PMC98999

[b20] Wozniak G. G. & Strahl B. D. Hitting the ‘mark’: Interpreting lysine methylation in the context of active transcription. Biochim. Biophys. Acta 1839, 1353–1361, doi: 10.1016/j.bbagrm.2014.03.002 (2014). 24631869

[b21] Wolffe A. P. & Pruss D. Targeting chromatin disruption: Transcription regulators that acetylate histones. Cell 84, 817–819, doi: 10.1016/s0092-8674(00)81059-4 (1996). 8601304

[b22] Spencer T. E. . Steroid receptor coactivator-1 is a histone acetyltransferase. Nature 389, 194–198 (1997). 9296499 10.1038/38304

[b23] Zhang Y. & Reinberg D. Transcription regulation by histone methylation: interplay between different covalent modifications of the core histone tails. Genes. Dev. 15, 2343–2360, doi: 10.1101/gad.927301 (2001). 11562345

[b24] Yang X. J. & Seto E. The Rpd3/Hda1 family of lysine deacetylases: from bacteria and yeast to mice and men. Nat. Rev. Mol. Cell. Biol. 9, 206–218, doi: 10.1038/nrm2346 (2008). 18292778 PMC2667380

[b25] Balasubramanyam K. . Curcumin, a novel p300/CREB-binding protein-specific inhibitor of acetyltransferase, represses the acetylation of histone/nonhistone proteins and histone acetyltransferase-dependent chromatin transcription. J. Biol. Chem. 279, 51163–51171, doi: 10.1074/jbc.M409024200 (2004). 15383533

[b26] Hatziapostolou M. . An HNF4 alpha-miRNA Inflammatory Feedback Circuit Regulates Hepatocellular Oncogenesis. Cell 147, 1233–1247, doi: 10.1016/j.cell.2011.10.043 (2011). 22153071 PMC3251960

[b27] Wang Z. Y. & Burke P. A. The role of microRNAs in hepatocyte nuclear factor-4alpha expression and transactivation. Biochim. Biophys. Acta 1829, 436–442, doi: 10.1016/j.bbagrm.2012.12.009 (2013). 23298640 PMC3625485

[b28] Zhang Y. X., Hagedorn C. H. & Wang L. Role of nuclear receptor SHP in metabolism and cancer. Biochim. Biophys. Acta 1812, 893–908, doi: 10.1016/j.bbadis.2010.10.006 (2011). 20970497 PMC3043166

[b29] Lee Y. K., Dell H., Dowhan D. H., Hadzopoulou-Cladaras M. & Moore D. D. The orphan nuclear receptor SHP inhibits hepatocyte nuclear factor 4 and retinoid X receptor transactivation: Two mechanisms for repression. Mol. Cell. Biol. 20, 187–195 (2000). 10594021 10.1128/mcb.20.1.187-195.2000 PMC85074

[b30] Morin P. J. & Storey K. B. Evidence for a reduced transcriptional state during hibernation in ground squirrels. Cryobiology 53, 310–318, doi: 10.1016/j.cryobiol.2006.08.002 (2006). 16979617

[b31] Hittel D. & Storey K. B. Differential expression of adipose- and heart-type fatty acid binding proteins in hibernating ground squirrels. Biochim. Biophys. Acta 1522, 238–243, doi: 10.1016/s0167-4781(01)00338-4 (2001). 11779641

[b32] Yan J., Barnes B. M., Kohl F. & Marr T. G. Modulation of gene expression in hibernating arctic ground squirrels. Physiol. Genomics 32, 170–181, doi: 10.1152/physiolgenomics.00075.2007 (2008). 17925484

[b33] Williams D. R. . Seasonally hibernating phenotype assessed through transcript screening. Physiol. Genomics 24, 13–22, doi: 10.1152/physiolgenomics.00301.2004 (2005). 16249311

[b34] Srere H. K., Wang L. C. H. & Martin S. L. Central role for differential gene-expression in mammalian hibernation. Proc. Natl. Acad. Sci. USA 89, 7119–7123, doi: 10.1073/pnas.89.15.7119 (1992). 1379733 PMC49657

[b35] Hampton M. . Deep Sequencing the Transcriptome Reveals Seasonal Adaptive Mechanisms in a Hibernating Mammal. Plos One 6, 13, doi: 10.1371/journal.pone.0027021 (2011). PMC3203946 22046435

[b36] Boyer B. B., Barnes B. M., Lowell B. B. & Grujic D. Differential regulation of uncoupling protein gene homologues in multiple tissues of hibernating ground squirrels. Am. J. Physiol. 275, R1232–R1238 (1998). 9756555 10.1152/ajpregu.1998.275.4.R1232

[b37] O’Hara B. F. . Gene expression in the brain across the hibernation cycle. Am. J. Physiol. 19, 3781–3790 (1999). 10.1523/JNEUROSCI.19-10-03781.1999 PMC6782720 10234010

[b38] Epperson L. E. & Martin S. L. Quantitative assessment of ground squirrel mRNA levels in multiple stages of hibernation. Physiol. Genomics 10, 93–102, doi: 10.1152/physiolgenomics.00004.2002 (2002). 12181366

[b39] Fujii G. . CpG methylation at the USF-binding site is important for the liver-specific transcription of the chipmunk HP-27 gene. Biochem. J. 395, 203–209, doi: 10.1042/bj20051802 (2006). 16396632 PMC1409699

[b40] Wang Z. B. . Genome-wide Mapping of HATs and HDACs Reveals Distinct Functions in Active and Inactive Genes. Cell 138, 1019–1031, doi: 10.1016/j.cell.2009.06.049 (2009). 19698979 PMC2750862

[b41] Ram O. . Combinatorial Patterning of Chromatin Regulators Uncovered by Genome-wide Location Analysis in Human Cells. Cell 147, 1628–1639, doi: 10.1016/j.cell.2011.09.057 (2011). 22196736 PMC3312319

[b42] Berger S. L. The complex language of chromatin regulation during transcription. Nature 447, 407–412, doi: 10.1038/nature05915 (2007). 17522673

[b43] Johansson L. . The orphan nuclear receptor SHP utilizes conserved LXXLL-related motifs for interactions with ligand-activated estrogen receptors. Mol. Cell. Biol. 20, 1124–1133, doi: 10.1128/mcb.20.4.1124-1133.2000 (2000). 10648597 PMC85230

[b44] Kim Y. C. . Farnesoid X receptor-induced lysine-specific histone demethylase reduces hepatic bile acid levels and protects the liver against bile acid toxicity. Hepatology 62, 220–231, doi: 10.1002/hep.27677 (2015). 25545350 PMC4480214

[b45] Mulligan P. . A SIRT1-LSD1 Corepressor Complex Regulates Notch Target Gene Expression and Development. Mol. Cell 42, 689–699, doi: 10.1016/j.molcel.2011.04.020 (2011). 21596603 PMC3119599

[b46] van Breukelen F. & Martin S. L. Reversible depression of transcription during hibernation. J. Comp. Physiol. B. 172, 355–361, doi: 10.1007/s00360-002-0256-1 (2002). 12122451

[b47] Van Breukelen F. & Martin S. L. Translational initiation is uncoupled from elongation at 18 degrees C during mammalian hibernation. Am. J. Physiol. Regul. Integr. Comp. Physiol. 281, R1374–R1379 (2001). 11641105 10.1152/ajpregu.2001.281.5.R1374

[b48] Osborne P. G., Gao B. & Hashimoto M. Determination *in vivo* of newly synthesized gene expression in hamsters during phases of the hibernation cycle. Jpn. J. Physiol. 54, 295–305, doi: 10.2170/jjphysiol.54.295 (2004). 15541207

[b49] Seldin M. M. . Seasonal oscillation of liver-derived hibernation protein complex in the central nervous system of non-hibernating mammals. J. Exp. Biol. 217, 2667–2679, doi: 10.1242/jeb.095976 (2014). 25079892 PMC4117459

[b50] Fujita S. . Identification of bovine hibernation-specific protein complex and evidence of its regulation in fasting and aging. J. Biochem. 153, 453–461, doi: 10.1093/jb/mvt008 (2013). 23389309

[b51] Hut R. A., Dardente H. & Riede S. J. Seasonal Timing: How Does a Hibernator Know When to Stop Hibernating? Curr. Biol. 24, R602–R605, doi: 10.1016/j.cub.2014.05.061 (2014). 25004363

[b52] de Miera C. S. . A Circannual Clock Drives Expression of Genes Central for Seasonal Reproduction. Curr. Biol. 24, 1500–1506, doi: 10.1016/j.cub.2014.05.024 (2014). 24980500

