# Supplementary material for: HNF-4 participates in the hibernation-associated transcriptional regulation of the chipmunk hibernation-related protein gene
Source: Sci Rep. 2017 Mar 10;7:44279. doi: 10.1038/srep44279 (PMC5345028; doi:10.1038/srep44279)
Supplement: Supplementary Information [file srep44279-s1.pdf]

Supplementary Information

HNF-4 participates in the hibernation-associated transcriptional regulation of the chipmunk hibernation-related protein gene

Daisuke Tsukamoto[1], Michihiko Ito[1] & Nobuhiko Takamatsu[1]

[1]Kitasato University School of Science, Kanagawa 252-0373, Japan. Correspondence and requests for materials should be addressed to D.T. (email: tsukamot@kitasato-u.ac.jp) or N.T. (email: takamatu@sci.kitasato-u.ac.jp)

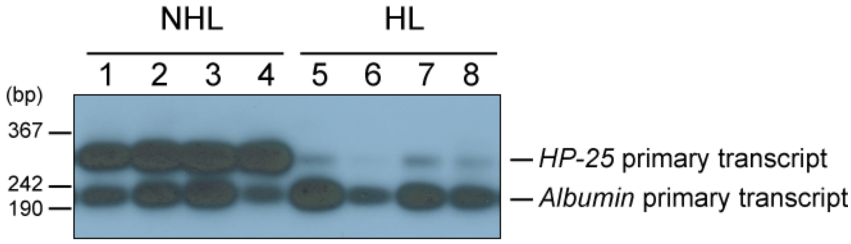

Supplementary Figure S1. *HP-25* is regulated at the transcriptional level in association with hibernation; Related to Figure 1

HP-25 and *albumin* primary transcripts were amplified by RT-PCR using total RNA extracted from the liver of nonhibernating (NHL; lanes 1-4) and hibernating chipmunks (HL; lanes 5-8). The PCR products were separated by electrophoresis on a 2% agarose gel, transferred to a nylon membrane, and hybridized with ^{32}P-labeled *HP-25*- and *albumin*-specific synthetic oligonucleotides probes. The oligonucleotides used for hybridization were CM25+280F, 5’-AGTGCCCATAACTCTGTCAA-3’; CMALB+961F, 5’-TATTCTCTCCTGTCATCAGG-3’.

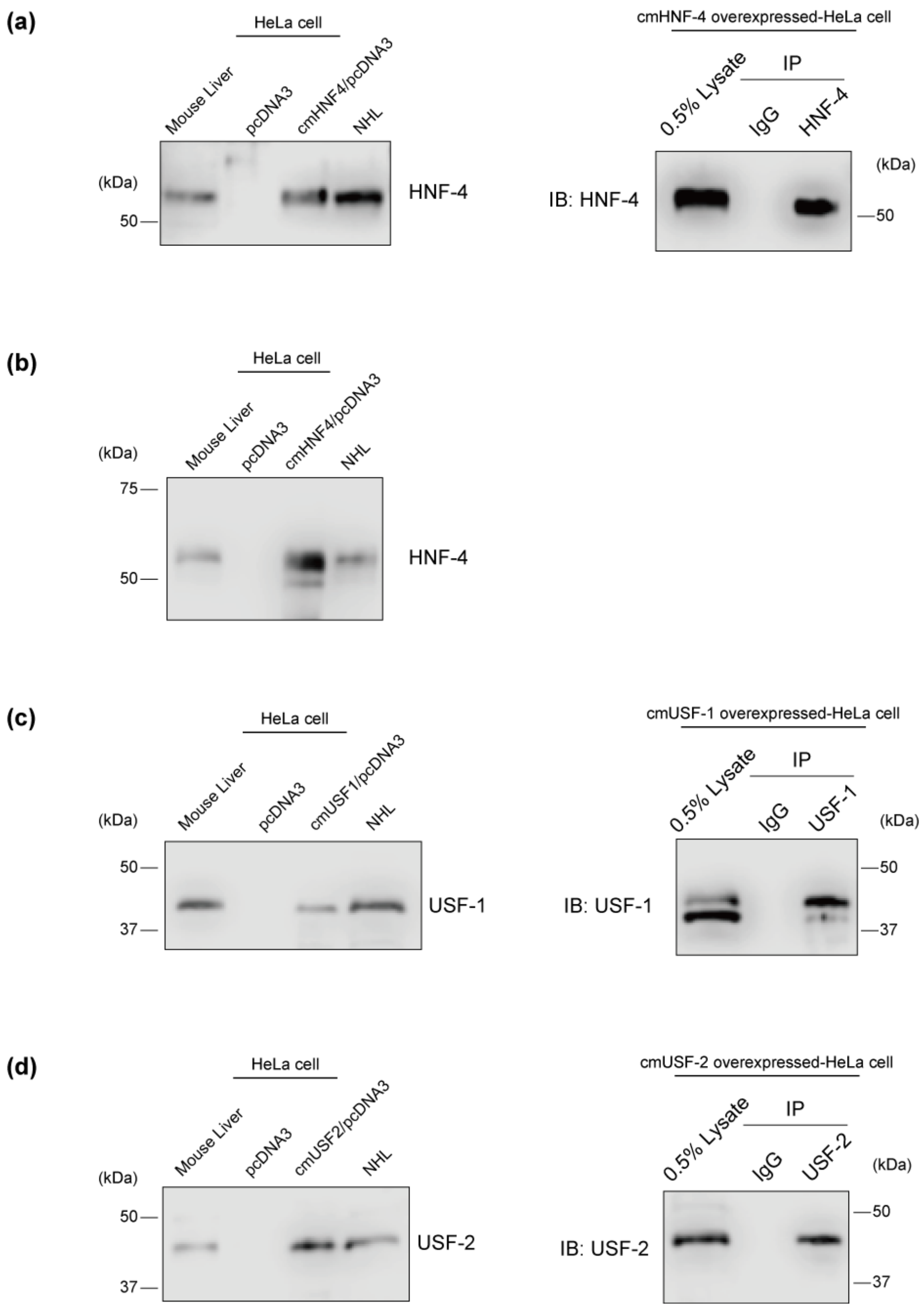

Supplementary Figure S2. Antibodies can recognize chipmunk proteins; Related to Figure 2

Antibodies against HNF-4 (Santa Cruz Biotechnology, sc-8987) (a), HNF-4 (Perseus Proteomics, H1415) (b), USF-1 (Santa Cruz Biotechnology, sc-229) (c) and USF-2 (Santa Cruz Biotechnology, sc-862) (d) were evaluated by western blotting (WB) (left panels) and immunoprecipitation (IP) (right panels). In WB, lysates of HeLa cells transfected with pcDNA3 or the corresponding chipmunk expression plasmid, lysates of mouse liver, and the nuclear extracts of a nonhibernating chipmunk liver (NHL) were used. In IP, the same HeLa cell lysates as in WB were used.

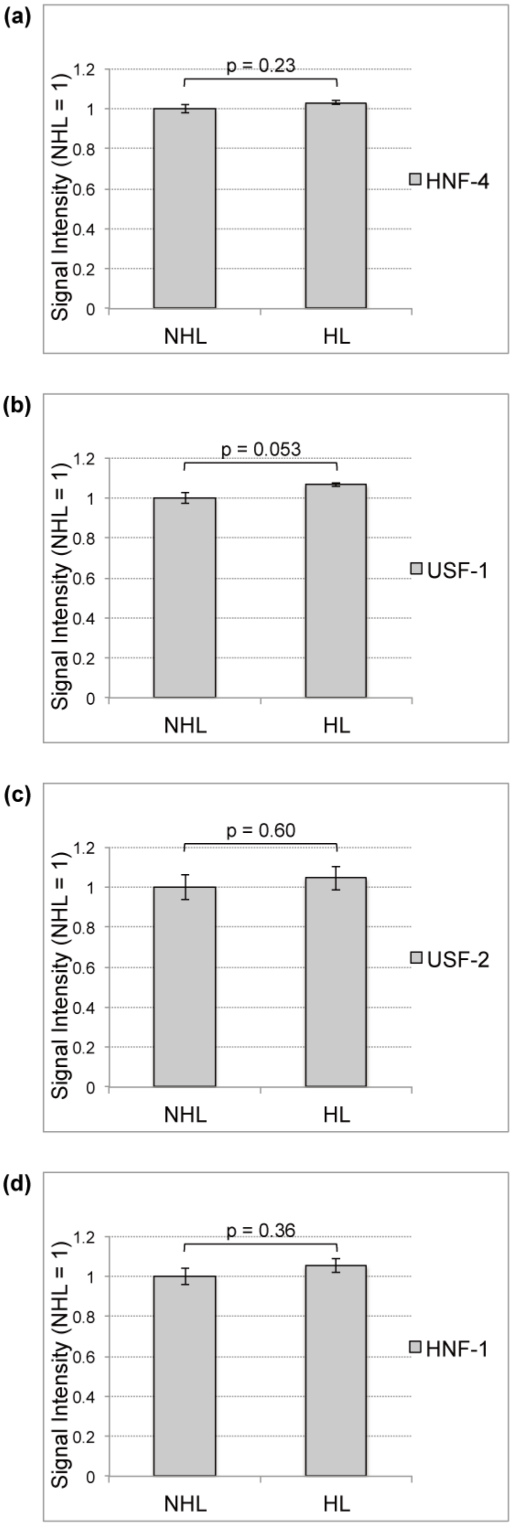

Supplementary Figure S3. Quantification of chemiluminescent western blotting bands; Related to Figure 2b

Signal quantification of western blot anaylysis in fig.2b. was performed using acquired gel images on C-DiGit Blot scanner with Image Studio 4.0 software (LI-COR Biosciences UK Ltd, Cambridge, UK). Relative signal values of HNF-4 (a), USF-1 (b), USF-2 (c), and HNF-1 (d) were normalized to the NHL average value. Results are means ± SEM. Student's t-test (2-tailed 2, type 2) was used to compare the difference.

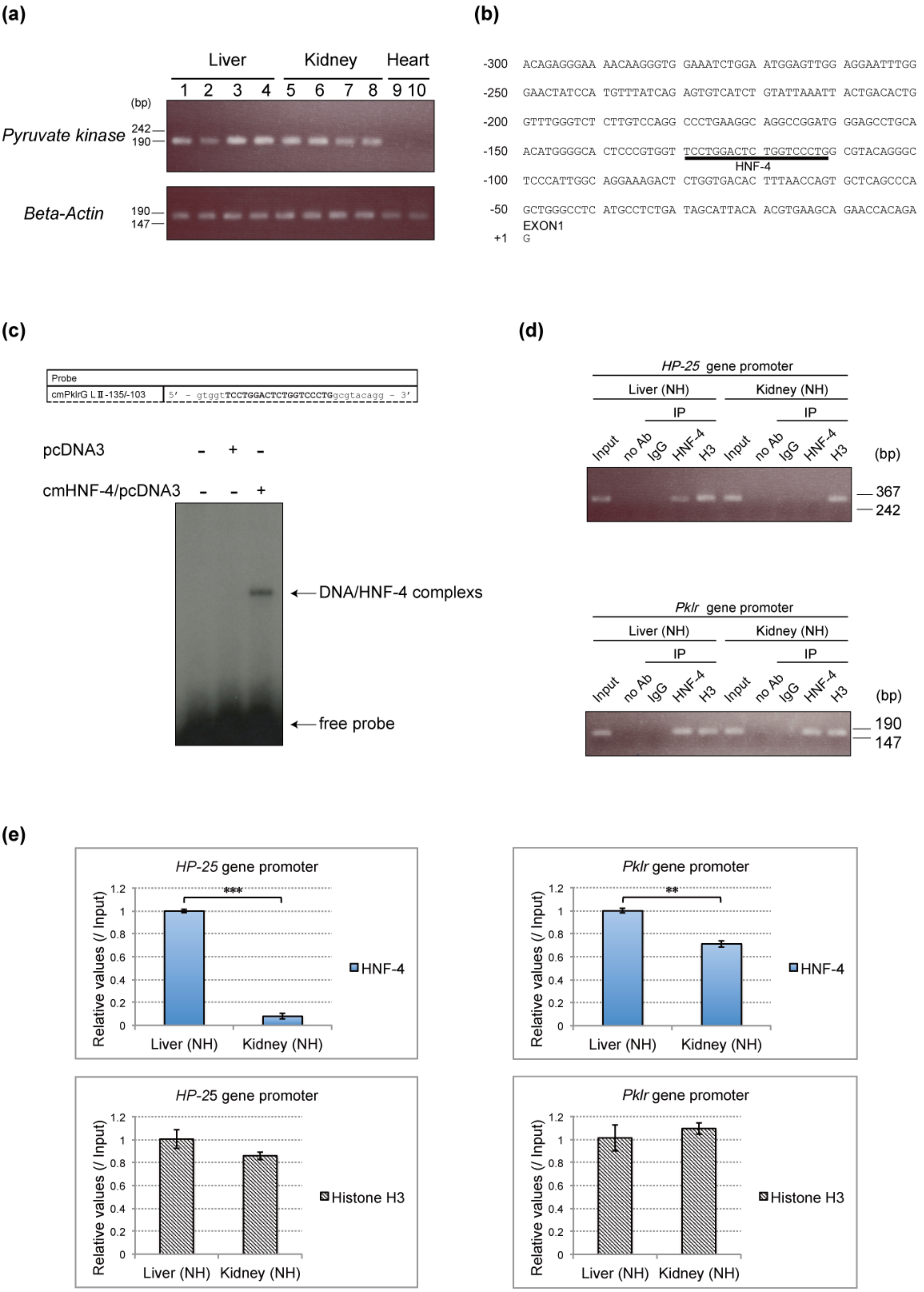
(a)
Liver
Kidney
Heart
1 2 3 4 5 6 7 8 9 10
(bp)
Pyruvate kinase
242
190
Beta-Actin
190
147
(b)
-300 ACAGAGGGAA AACAAGGGTG GAAATCTGGA ATGGAGTTGG AGGAATTTGG
-250 GAACTATCCA TGTTTATCAG AGTGTCATCT GTATTAAATT ACTGACACTG
-200 GTTTGGGTCT CTTGTCCAGG CCCTGAAGGC AGGCCGGATG GGAGCCTGCA
-150 ACATGGGGCA CTCCCGTGGT TCCTGGACTC TGGTCCCTGG CGTACAGGGC
HNF-4
-100 TCCCATTGGC AGGAAAGACT CTGGTGACAC TTTAACCAGT GCTCAGCCCA
-50 GCTGGGCCTC ATGCCTCTGA TAGCATTACA ACGTGAAGCA GAACCACAGA
EXON1
+1 G
(c)
Probe
cmPklrG LⅡ-135/-103
5' - gtggtTCCTGGACTCTGGTCCCTGgcgtacagg - 3'
pcDNA3 - + -
cmHNF-4/pcDNA3 - - +
DNA/HNF-4 complexs
free probe
(d)
HP-25 gene promoter
Liver (NH)
Kidney (NH)
IP
Input no Ab IgG HNF-4 H3
(bp)
367
242
Pklr gene promoter
Liver (NH)
Kidney (NH)
IP
Input no Ab IgG HNF-4 H3
(bp)
190
147
(e)
HP-25 gene promoter
Relative values (/ Input)
***
HNF-4
Liver (NH) Kidney (NH)
Pklr gene promoter
**
HNF-4
HP-25 gene promoter
Histone H3
Pklr gene promoter
Histone H3
0 0.2 0.4 0.6 0.8 1 1.2

Supplementary Figure S4. HNF-4 binds to the *HP-25* gene promoter liver-specifically; Related to Figure 2c

(a) RT-PCR analysis of *pyruvate kinase* (*Pklr*) gene in chipmunk liver and kidney. cDNA was synthesized with total RNA prepared from liver, kidney, or heart of four nonhibernating chipmunks, and *Pklr* and *beta-actin* cDNA was amplified by PCR. (b) Nucleotide sequence of the chipmunk *Pklr* gene promoter region. The putative transcription start site is indicated as +1. Potential HNF-4 binding site is indicated below the sequence. (c) EMSA of binding of *in vitro* translated chipmunk HNF-4 to the *Pklr* gene promoter. (d) ChIP performed with chromatin from liver and kidney of a nonhibernating chipmunk using the indicated antibodies. (e) ChIP-qPCR performed using the same samples as in (d). The values were normalized to the total input values, and the results are shown as the fold increase over the values for the Liver (NH). Results are means ± SEM for one experiment performed in triplicate. $^{**}p < 0.01$, $^{***}p < 0.001$.

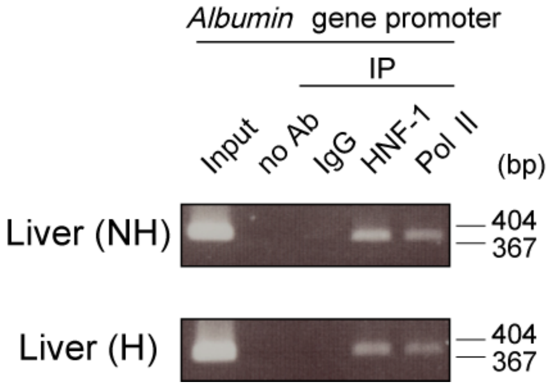

Supplementary Figure S5. HNF-1 and Pol II bound to the albumin gene promoter region in the liver of both nonhibernating and hibernating chipmunks; Related to Figure 2c

ChIP assays were performed with chromatin from the liver of a nonhibernating chipmunk (NH) and a hibernating chipmunk (H) using the indicated antibodies, normal rabbit IgG (IgG), or no antibody (no Ab). Following DNA purification, the samples were analyzed by PCR using primer set specific for the albumin gene promoter region. One hundredth of four percent of the total input sample (Input) was also examined by PCR. Results are representative of more than three experiments.

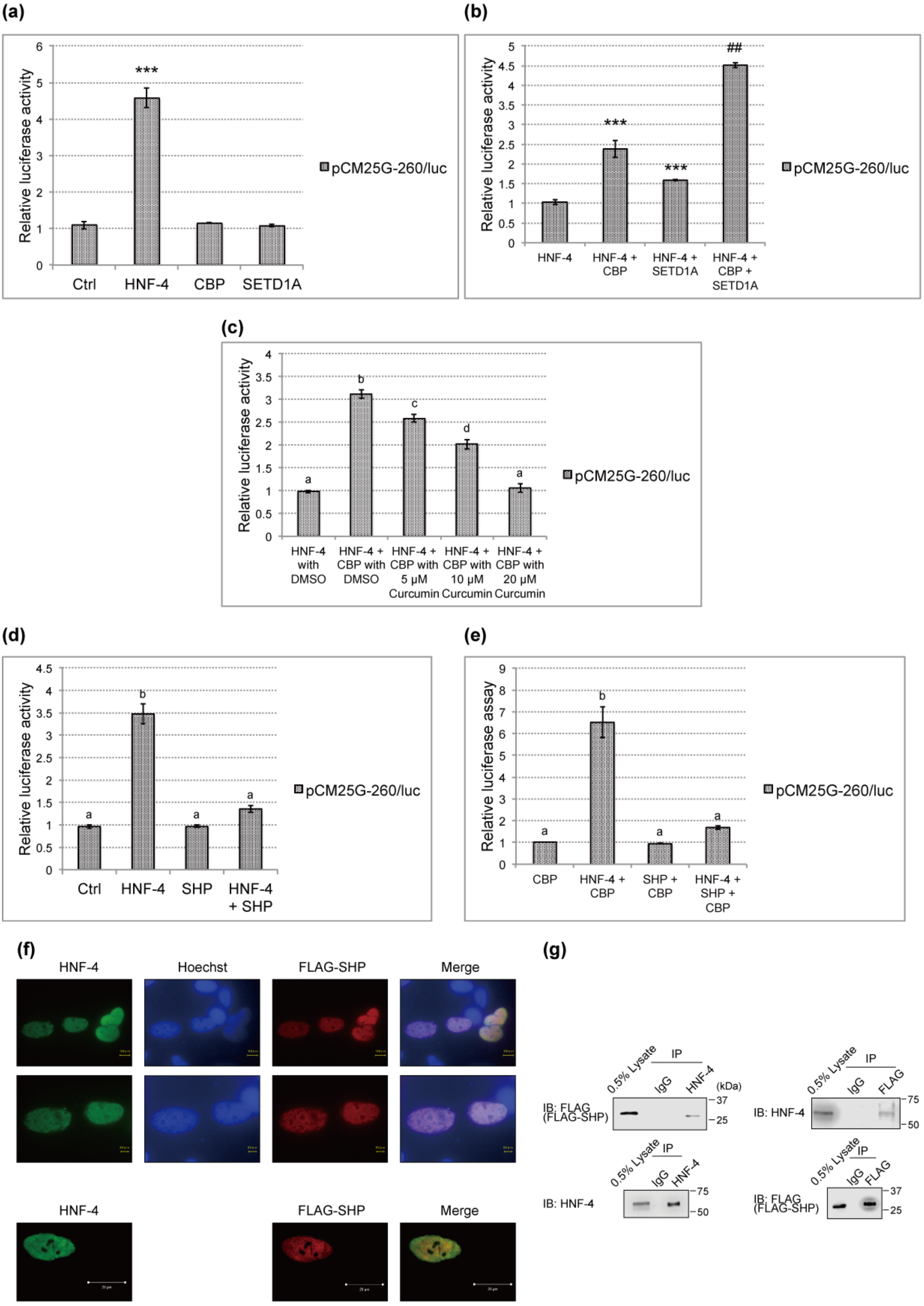
(a)
Relative luciferase activity
0
1
2
3
4
5
6
***
Ctrl
HNF-4
CBP
SETD1A
pCM25G-260/luc
(b)
Relative luciferase activity
0
0.5
1
1.5
2
2.5
3
3.5
4
4.5
5
***
***
##
HNF-4
HNF-4 + CBP
HNF-4 + SETD1A
HNF-4 + CBP + SETD1A
pCM25G-260/luc
(c)
Relative luciferase activity
0
0.5
1
1.5
2
2.5
3
3.5
4
a
b
c
d
a
HNF-4 with DMSO
HNF-4 + CBP with DMSO
HNF-4 + CBP with 5 μM Curcumin
HNF-4 + CBP with 10 μM Curcumin
HNF-4 + CBP with 20 μM Curcumin
pCM25G-260/luc
(d)
Relative luciferase activity
0
0.5
1
1.5
2
2.5
3
3.5
4
4.5
a
b
a
a
Ctrl
HNF-4
SHP
HNF-4 + SHP
pCM25G-260/luc
(e)
Relative luciferase assay
0
1
2
3
4
5
6
7
8
9
a
b
a
a
CBP
HNF-4 + CBP
SHP + CBP
HNF-4 + SHP + CBP
pCM25G-260/luc
(f)
HNF-4
Hoechst
FLAG-SHP
Merge
HNF-4
FLAG-SHP
Merge
(g)
0.5% Lysate
IP
IgG
HNF-4
(kDa)
IB: FLAG (FLAG-SHP)
37
25
0.5% Lysate
IP
IgG
FLAG
IB: HNF-4
75
50
0.5% Lysate
IP
IgG
HNF-4
IB: HNF-4
75
50
0.5% Lysate
IP
IgG
FLAG
IB: FLAG (FLAG-SHP)
37
25

Supplementary Figure S6. The effects of the various cofactors on the HNF-4 transcriptional activity in HeLa cells; Related to Figure 4 and 5.

(a-e) HeLa cells were transfected with expression constructs for HNF-4, CBP, SETD1A, and/or SHP, together with the *HP-25* gene promoter-reporter construct pCM25G-260/luc and the internal control plasmid pRL-SV40. In (c), the indicated concentration of curcumin or DMSO (solvent control) was added to the medium. Firefly luciferase activity was normalized to *Renilla* luciferase activity, and the data are shown as the fold increase over the luciferase activity of pCM25G-260/luc alone (Ctrl) (a, d), pCM25G-260/luc plus HNF-4 (b), with DMSO (c), or pCM25G-260/luc plus CBP (e). Results show means ± SEM for three (a, b, d, e) or two (c) independent experiments performed in technical triplicate. In (a), ***$p < 0.001$, compared with Ctrl; Tukey-Kramer test. In (b), ***$p < 0.001$, compared with pCM25G-260/luc plus HNF-4; two-way ANOVA (##$p < 0.01$ for interaction; two-way ANOVA). In (c), Different letters (a-d) are significantly different at $p < 0.01$; Tukey-Kramer test. In (d,e), Different letters (a,b) are significantly different at $p < 0.001$; two-way ANOVA with Tukey-Kramer post test. (f) Immunocytochemistry was performed using HeLa cells transfected with HNF-4 and FLAG-SHP. The cells were observed by fluorescence microscopy (upper and middle panels) or confocal microscopy (lower panels). Data shown are representative cells from one of three independent experiments. Hoechst: the DNA-binding dye Hoechst 33258. Scale bar: 10 µm (upper panels), 5 µm (middle panels), and 20 µm (lower panels). (g) Protein extracts from HeLa cells transfected with HNF-4 and FLAG-SHP were immunoprecipitated (IP) using an anti-HNF-4 antibody (left panels), an anti-FLAG antibody (right panels), or IgG, and the immunoprecipitated samples were immunoblotted (IB) with the indicated antibodies. The presence of HNF-4 and FLAG-SHP in 0.5% total lysate was verified by immunoblotting with specific antibodies.
